# Increased PRAME-Specific CTL Killing of Acute Myeloid Leukemia Cells by Either a Novel Histone Deacetylase Inhibitor Chidamide Alone or Combined Treatment with Decitabine

**DOI:** 10.1371/journal.pone.0070522

**Published:** 2013-08-05

**Authors:** Yushi Yao, Jihao Zhou, Lixin Wang, Xiaoning Gao, Qiaoyang Ning, Mengmeng Jiang, Jia Wang, Lili Wang, Li Yu

**Affiliations:** 1 Department of Hematology and BMT Center, Chinese PLA General Hospital, Beijing, China; 2 Department of Hematology, Navy General Hospital, Beijing, China; European Institute of Oncology, Italy

## Abstract

As one of the best known cancer testis antigens, PRAME is overexpressed exclusively in germ line tissues such as the testis as well as in a variety of solid and hematological malignant cells including acute myeloid leukemia. Therefore, PRAME has been recognized as a promising target for both active and adoptive anti-leukemia immunotherapy. However, in most patients with PRAME-expressing acute myeloid leukemia, PRAME antigen-specific CD8^+^ CTL response are either undetectable or too weak to exert immune surveillance presumably due to the inadequate PRAME antigen expression and PRAME-specific antigen presentation by leukemia cells. In this study, we observed remarkably increased PRAME mRNA expression in human acute myeloid leukemia cell lines and primary acute myeloid leukemia cells after treatment with a novel subtype-selective histone deacetylase inhibitor chidamide *in vitro*. PRAME expression was further enhanced in acute myeloid leukemia cell lines after combined treatment with chidamide and DNA demethylating agent decitabine. Pre-treatment of an HLA-A0201^+^ acute myeloid leukemia cell line THP-1 with chidamide and/or decitabine increased sensitivity to purified CTLs that recognize PRAME^100–108^ or PRAME^300–309^ peptide presented by HLA-A0201. Chidamide-induced epigenetic upregulation of CD86 also contributed to increased cytotoxicity of PRAME antigen-specific CTLs. Our data thus provide a new line of evidence that epigenetic upregulation of cancer testis antigens by a subtype-selective HDAC inhibitor or in combination with hypomethylating agent increases CTL cytotoxicity and may represent a new opportunity in future design of treatment strategy targeting specifically PRAME-expressing acute myeloid leukemia.

## Introduction

Cancer testis antigens (CTAs) are a group of tumor-associated antigens that are expressed predominantly in germ line tissues such as the testis and malignant cells [1], [2]. Preferentially Expressed Antigen of Melanoma (PRAME) is one of the most studied CTAs that is over-expressed in a variety of solid and hematological malignant cells but is not or minimally expressed in normal non-germ line cells [3], [4], [5], [6], [7]. Due to its highly tumor-specific expression pattern and more importantly, its well defined immunogenicity as demonstrated by specific killing of PRAME-expressing leukemia cells by PRAME antigen-specific CTL clones, PRAME has been recognized as a promising target for anti-leukemia immunotherapy [8], [9], [10]. To date, 4 HLA-A0201-restricted PRAME epitopes have been identified: PRA^100–108^(VLDGLDVLL), PRA^142–151^(SLYSFPEPEA), PRA^300–309^(ALYVDSLFFL), and PRA^425–433^(SLLQHLIGL) [9], [11], which facilitated the detection and functional analysis of PRAME antigen-specific CTL responses both *in vitro* and *ex vivo* [12], [13].

Both histone deacetylase (HDAC) inhibitors and hypomethylating agents are being investigated as anti-tumor drugs [14], [15]. It has been reported that these two types of epigenetic modulators not only has direct anti-leukemia effects via inducing leukemia cell apoptosis and cell cycle arrest, but also boost anti-leukemia immune responses of T cells and NK cells via mechanisms involving upregulation of tumor associated antigens, MHC molecules, costimulatory molecules, adhesion molecules and ligands of NK cell activation receptors on leukemia cells [14], [16], [17], [18], [19], [20], [21], [22], [23], [24]. Expression of CTAs including PRAME is epigenetically regulated by DNA methylation [3], [6]. Mengyong Yan *et al.* reported an increased PRAME antigen-specific CTL killing of a variety of HLA-A0201^+^ hematological and solid tumor cell lines via decitabine induced upregulation of PRAME in these tumor cells [10]. Oliver Goodyear *et al.* reported an increased expression of MAGE-A1 mRNA and protein in acute myeloid leukemia (AML) cell lines after treatment with another hypomethylating agent azacitidine (AZA) alone or in combination with the HDAC inhibitor valproic acid (VPA) [25]. Combined treatment with AZA and VPA increased MAGE-A1 antigen-specific CD8^+^ T cell response in patients with AML or MDS, indicating antigen-specific immune activation. In Goodyear’s study, VPA treatment alone was not effective in upregulating MAGE-A1 expression, whereas VPA augmented AZA-boosted expression of MAGE-A1 and possibly other CTAs [25]. These data collectively pointed to a hypothesis that HDAC inhibitors and hypomethylating agents, administered alone or in combination in patients with leukemia, may enhance anti-leukemia T cell immunity via mechanisms including the upregulation of CTAs in leukemia cells [26]. However, there are controversial implications from different studies on respective roles in immunomodulation by individual HDAC inhibitors, i.e., effects on NK cytotoxicity, regulatory T cell activity, or dendritic cell functions [27], [28], [29], [30]. Thus, it is important to test further the potential immune regulatory property associated with different chemical class of HDAC inhibitors.

In this study, we treated AML cells *in vitro* with a novel benzamide chemical class of HDAC inhibitor chidamide (Epidaza, CS055) that selectively inhibited HDAC1, 2, 3 and 10, which is currently in phase II clinic developments against relapsed and refractory peripheral T cell lymphomas and non-small cell lung carcinomas in China and US [18], [31]. We observed significantly increased PRAME mRNA expression in AML cell lines and blast-containing bone marrow mononuclear cells from AML patients induced by chidamide but not in normal bone marrow or peripheral blood cells. In consistent with previous results, HDAC inhibition induced by either chidamide or VPA upregulated costimulatory molecule CD86 expression in AML cell lines [32]. HLA-I and CD80 on AML cell surface were not altered after treatment with chidamide or VPA. CTLs specific for 2 HLA-A0201-restricted PRAME epitopes (PRA^100–108^ and PRA^300–309^) were generated from healthy donors and their cytotoxicity against the HLA-A0201^+^ AML cell clone THP-1 was determined. After treatment of THP-1 cells with chidamide, significantly increased CTL mediated cytotoxicity was observed together with increased PRAME mRNA expression. Upregulation of CD86 contributed partly to this increased cytotoxicity. Though low dose decitabine alone was not effective in stimulating PRAME expression, it significantly increased chidamide induced upregulation of PRAME. In accordance with PRAME expression level, combined treatment of THP-1 with chidamide and decitabine further enhanced significantly the increased PRAME-specific CTL killing when compared with chidamide treatment alone. Pre-treatment of CTLs with chidamide, alone or in combination with decitabine, did not impair IFN-γ expression nor cytotoxic functions of CTLs. Taken together, our data showed an increased PRAME antigen-specific CTL cytotoxicity targeting AML cells after treatment with subtype selective HDAC inhibitor chidamide alone or in combination with hypomethylating agent decitabine *in vitro*, through mechanisms including the upregulation of PRAME and CD86 in AML cells.

## Materials and Methods

### Cell Lines and Reagents

Human acute myeloid leukemia (AML) cell lines Kasumi-1, K562, NB4, THP-1 and U937, human acute lymphoblastic leukemia (ALL) cell lines Hut78, Molt-4 and Z138 were purchased from Cell Culture Center of Peking Union Medical College (Beijing, China). T2 (TAP-deficient lymphoblastoid cell line) and SW480 (human colon carcinoma) cell lines were kindly provided by Professor Xuetao Cao (Chinese Academy of Medical Sciences). Cells were cultured with RPMI 1640 (SW480 was cultured with Leibovitz L-15 in an air incubator; both culture media were purchased from Hyclone) supplemented with 10% fetal bovine serum (FBS, purchased from Hyclone) penicillin and streptomycin at 37°C in a CO_2_ incubator. Chidamide was provided by Shenzhen Chipscreen Biosciences. Ltd. (Shenzhen, China), valproic acid (VPA) was purchased from Sigma-Aldrich. Decitabine was purchased from Xi’an Jansson Pharmaceutical Ltd. Purified anti-human HLA-A2 antibody and purified anti-human CD86 antibody for blockade, purified anti-human CD3 and purified anti-human CD28 for stimulating T cell proliferation were purchased from Biolegend. Recombinant human Interleukin-2 (rhIL-2), Interleukin-4 (rhIL-4), granulocyte-monocyte colony stimulating factor (rhGM-CSF), and Tumor Necrosis Factor-α (rhTNF-α) were purchased from Peprotech.

### Treatment with Chidamide, VPA and Decitabine

For HDAC inhibitor treatment, leukemia cell lines and bone marrow or peripheral blood mononuclear cells were treated with chidamide or VPA at the indicated concentrations for 24 h (and 48 h where specified) *in vitro*. For combined treatment with chidamide and decitabine, AML cell lines were treated with chidamide at 1 µM for 24 h, in combination to decitabine (250 nM) supplemented in culture media twice for 48 h at 24 h interval. Cells were then washed and harvested for analysis.

### Cell Cycle, Apoptosis and Colony Forming Assays

For cell cycle assay, cells were fixed in 70% ethanol at 4°C overnight, followed by incubation with 10 µg/ml Ribonuclease A (Sigma-Aldrich) at 37°C for 30 min. Cells were then incubated with 50 µg/ml propidium iodide (BD Biosciences) and cell cycle was analyzed on a FACScalibur flow cytometer (Becton Dickinson). ModFit LT software (Version 3.1, Verity Software House Inc., Topsham, ME, USA) was used for cell cycle analysis based on DNA content. For apoptosis assay, cells were stained with Annexin V-FITC (BD Biosciences) and propidium iodide, followed by FACS analysis. For colony forming assay, THP-1 cells were suspended in Methocult H4230 (STEMCELL) at 1×10^3^ cells/ml, plated in 24-well plate in triplicates and cultured for 7 to 14 days. The frequency of colony forming units (CFU) was calculated as number of colonies counted/well.

### Collection of Samples from Patients and Healthy Donors

Bone marrow and peripheral blood samples from healthy donors and bone marrow samples from AML patients were collected. Peripheral blood samples from two healthy donors that are HLA-A0201^+^ were collected for generation of PRAME epitope specific CTLs. Written informed consent was obtained from all patients and healthy donors. The use of clinical samples in our experiments was in accordance with the Declaration of Helsinki, and was approved by the Ethics Committee of Chinese PLA General Hospital.

### Peptides

PRA^100–108^(VLDGLDVLL) and PRA^300–309^(ALYVDSLFFL) were synthesized and HPLC purified to over 95% by Beijing SBS Genetech Co., Ltd. (Beijing, China).

### HLA-A Typing

HLA-A typing of leukemia cell lines and healthy donors were screened by a PCR-based analysis, as well as by FACS analysis using FITC-conjugated anti-HLA-A2 mAb to identify possible HLA-A0201^+^ cell lines and donors. To confirm HLA-A0201 expression in two healthy donors and the AML cell line THP-1, DNA sequencing-based high-resolution HLA-A typing was conducted by Beijing Search Biotech (Beijing, China).

### Transient Transfection

Full length PRAME coding sequence (1,930 bp, cloned from cDNA of K562 cells using the following forward and reverse primers: CCCAAGCTTATGGAACGAAGGCGTTTGTGG and CCGGAATTCCTAGTTAGGCATGAAACAGGG) was cloned into pcDNA3.0 vector. We transfected PRAME or empty vector into a PRAME negative HLA-A0201^+^ cell line SW480 using Superfect reagent(Qiagen) according to manufacturer’s instruction [10]. The transfection efficiency was 70–85% as demonstrated by co-transfer of eGFP vector followed by flow cytometry analysis.

### Quantitative-RT-PCR

RNA was extracted from cells and cDNA was obtained after reverse transcription. Expression of PRAME and GAPDH was quantified by SYBRgreen real-time quantitative PCR analysis on an Mx3000p light cycler (Stratagene), and data were analyzed using Mx3000p software. Primers for PRAME (forward and reverse): GCTGTGCTTGATGGACTTGA and ATTCATCACAGGCACCTTCC. Primers for GAPDH (forward and reverse): GAGTCAACGGATTTGGTCGT and TTGATTTTGGAGGGATCTCG. PRAME mRNA expression was expressed as 2^−ΔCT^ relative to GAPDH.

### Western Blot

Polyclonal rabbit anti-human PRAME and total histone H3 primary antibodies were purchased from ABcam. Polyclonal rabbit anti-human acetylated histone H3 and β-actin, monoclonal rabbit anti-human CDK2, monoclonal mouse anti-human CDK4 primary antibodies, and HRP-linked anti-rabbit IgG and anti-mouse IgG secondary antibodies were purchased from Cell Signaling.

### Flow Cytometry

For fluorescence activated cell sorting (FACS) analysis, recombinant Soluble Dimeric Human HLA-A2:Ig Fusion Protein, PE conjugated anti-mouse IgG1, and FITC conjugated mouse anti-human CD8 (G42-8) were purchased from BD PharMingen. Other fluorescence conjugated antibodies for flow cytometry were purchased from Biolegend or eBiosciences unless otherwise specified. For intracellular staining of IFN-γ and TNF-α, Golgistop (BD Biosciences) was added to culture medium 5 h before harvest at 1/1,000. Fluorescence labeled cells were analyzed on a BD FACSCalibur cytometer. Data were analyzed using Flowjo software version 7.6.1.(Tree Star).

### Generation of PRA^100–108^-HLA-A0201 and PRA^300–309^-HLA-A0201 Specific CTLs

We used a modified quick expansion protocol to generate PRAME specific CTLs [33]. Peripheral blood samples were collected from 2 healthy HLA-A0201^+^ donors. Mononuclear cells (PBMCs) were obtained by Ficoll-Paque gradient centrifuge. Adherent PBMCs were cultured in RPMI-1640 supplemented with 10% FBS, rhGM-CSF (800 U/ml) and rhIL-4 (500 U/ml) to generate dendritic cells (DCs). On day 6 of culture, DCs were matured with rhTNF-α (100 U/ml) for 24 h. Autologous DCs were pulsed with 10 µg/ml PRAME^100–108^ or PRAME^300–309^ peptides and cocultured with T cell-containing non-adherent cells at a DC:T = 1∶10 ratio in RPMI 1640 supplemented with 10% FBS, 50 U/ml of rhIL-2, HEPES and β-ME. After 2 cycles of stimulation at weekly intervals, CD3^+^CD8^+^PRAME-HLA-A0201 Dimer^+^ cells were FACS sorted using a Moflo XDP cell sorter (Beckman-Coulter), followed by a rapid expansion protocol using anti-CD3 and anti-CD28 stimulation at the presence of 50 U/ml rhIL-2 to generate PRAME specific CTLs.

### Cytotoxicity Assay

We used a FACS-based analysis to determine CTL killing of target cells [34]. Briefly, target cells were labeled with CFSE (5 µM) and cultured at 5,000 cells per well without CTLs (as spontaneous death control) or with PRAME specific CTLs at various E/T ratios (10/1, 20/1 or 40/1; where not specified, E/T ratio of 20/1 was used) in triplicates in 96-well U-bottom cell culture plates. To confirm the antigen specificity of PRAME antigen-specific CTLs in our experiments, T2 cells were pulsed with PRA^100–108^ peptide (1–10,000 nM) for 1 hour, washed once with culture medium and used as target cells. For cold target inhibition experiments, PRA^100–108^ peptide-pulsed T2 cells were used as cold targets. Ratios of 30/1 and 10/1 cold (T2) to hot (chidamide treated THP-1 cells, labeled with CFSE) target were used [12]. The 96-well plates were centrifuged and cultured at 37°C in a CO_2_ incubator for 24 hours, followed by PI staining of cells in each well and FACS analysis.
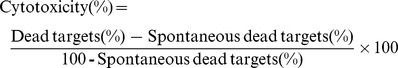



### Statistical Analysis

Data are presented as mean±S.D. Student *t*-test was used to compare data between groups. A value of *P*<0.05 was considered statistically significant.

## Results

### Upregulation of PRAME Expression in AML Cells after HDAC Inhibition

**Figure 1:**
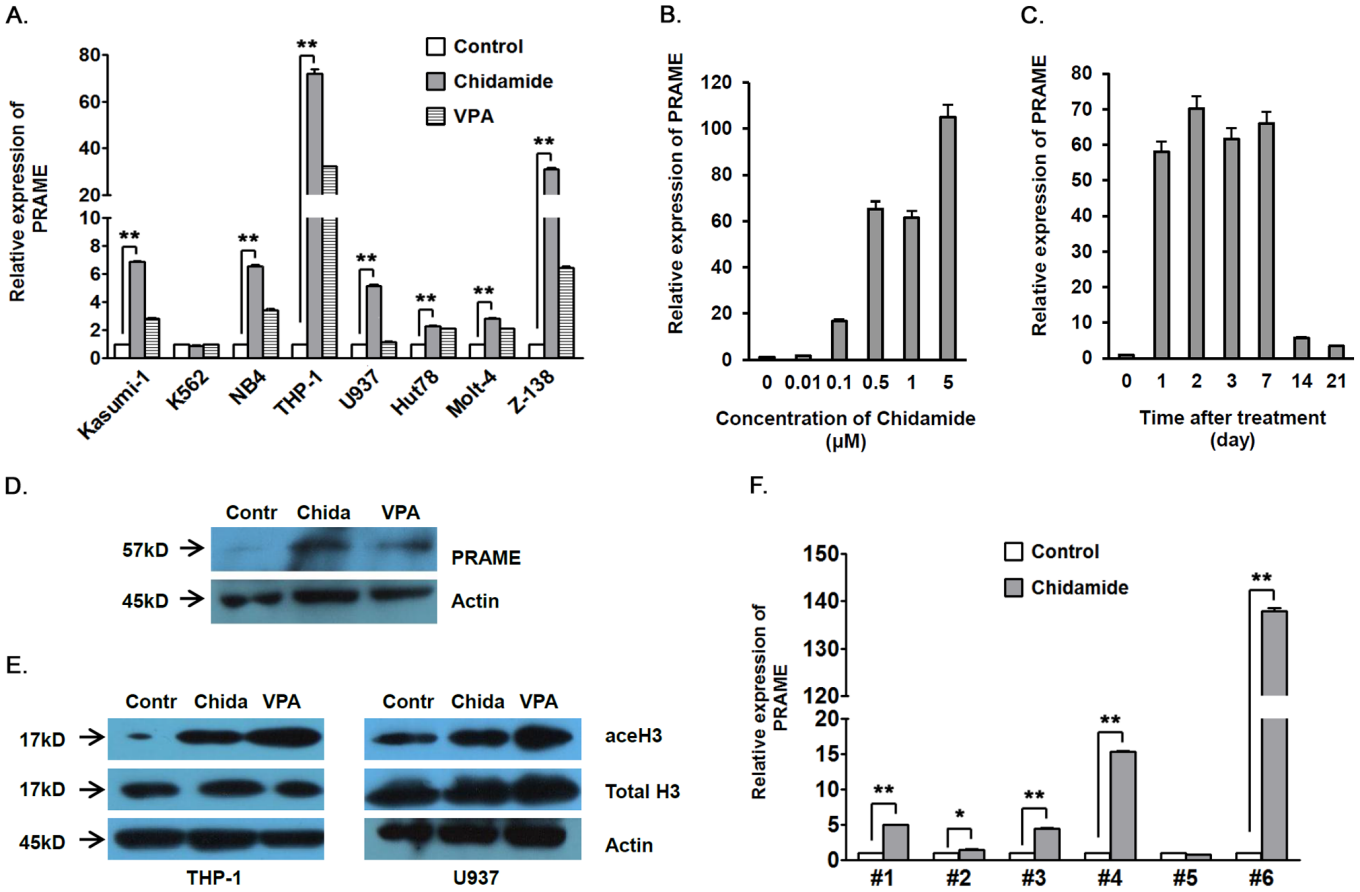
Upregulation of PRAME expression in human AML cells after HDAC inhibition. **A.** PRAME mRNA expression in human leukemia cell lines after chidamide of VPA treatment. Human AML cell lines Kasumi, NB4, U937, THP-1, and K562, ALL cell lines Hut78, Molt-4, and Z-138 were treated *in vitro* with chidamide at 1 µM or VPA at 1 mM for 24 h. PRAME mRNA expression was analyzed by real-time quantitative RT-PCR (SYBRgreen). **B.** THP-1 cells were treated *in vitro* with chidamide at various concentrations for 24 h. PRAME mRNA expression was determined by real-time quantitative RT-PCR. **C.** PRAME mRNA expression of THP-1 cells at various time points after chidamide treatment at 1 µM for 24 h. **D.** Western blot analysis of PRAME protein expression of THP-1 cells that were not treated or treated *in vitro* with chidamide at 1 µM or VPA at 1 mM for 48 h. **E.** H3 histone acetylation in untreated, chidamide treated, or VPA treated AML cell lines THP-1 and U937. **F.** Bone marrow cells from patients with PRAME-expressing AML were treated *ex vivo* with chidamide (1 µM for 24 h), followed by real-time quantitative RT-PCR analysis of PRAME mRNA. See Table1 for patient information. In D and E, Contr = Control; Chida = Chidamide. In A, B, C and F, Y axis shows folds of PRAME mRNA expression relative to that of non-treated cells. *P<0.05, **P<0.01.

We analyzed PRAME mRNA expression in AML cell lines Kasumi-1, K562, NB4, THP-1, and U937, ALL cell lines Hut78, Molt-4 and Z138 before and after treatment with HDAC inhibitors chidamide or VPA *in vitro*. The relative expression of PRAME mRNA was increased by around 5 to 75 folds in AML and 2 to 30 folds in ALL cell lines after treatment with chidamide, except for K562 cells that had very high baseline PRAME mRNA expression. Treatment with a classical pan-HDAC inhibitor VPA also increased PRAME mRNA expression (Figure 1A), though to a less extent. In contrast to leukemia cell lines, PRAME mRNA expression was not detected before or after *in vitro* chidamide treatment in peripheral blood or bone marrow mononuclear cells from two healthy donors (Figure S1). PRAME mRNA expression in THP-1 cells showed a dose dependent increase after chidamide treatment at concentrations from 0.01 to 5 µM (Figure 1B). After chidamide treatment, PRAME mRNA expression kept continuously upregulated for at least 1 week and started to decrease thereafter, while remaining higher than non-treated THP-1 cells 3 weeks after treatment (Figure 1C). In accordance with the upregulation of PRAME mRNA, western blot analysis showed increased PRAME protein expression in THP-1 cells after treatment with chidamide or VPA *in vitro* (Figure 1D and see Figure S6A, B for representative raw data). Acetylated histone H3 was higher in HDAC inhibitor treated THP-1 or U937 cells than non-treated cells (Figure 1E). In bone marrow mononuclear cells from 6 PRAME^+^ AML patients, *in vitro* treatment with chidamide upregulated PRAME mRNA expression in 5/6 bone marrow samples by around 1.5 to 138 folds. Minimally decreased PRAME expression was observed in 1 bone marrow sample after chidamide treatment. Since the percentage of leukemia blasts in all the bone marrow samples were not altered after chidamide treatment for 24 h (data not shown), we excluded the possibility that increased PRAME expression may derive from increased percentage of leukemia cells in bone marrow cells (Figure 1F and see Table1 for patient information). Thus, our data clearly showed increased PRAME expression in AML cells after treatment with HDAC inhibitor chidamide or VPA *in vitro*.

### Chidamide Induces Apoptosis, Cell Cycle Arrest, Reduces Colony Forming Ability, and Upregulates CD86 Expression in AML Cell Lines

**Figure 2:**
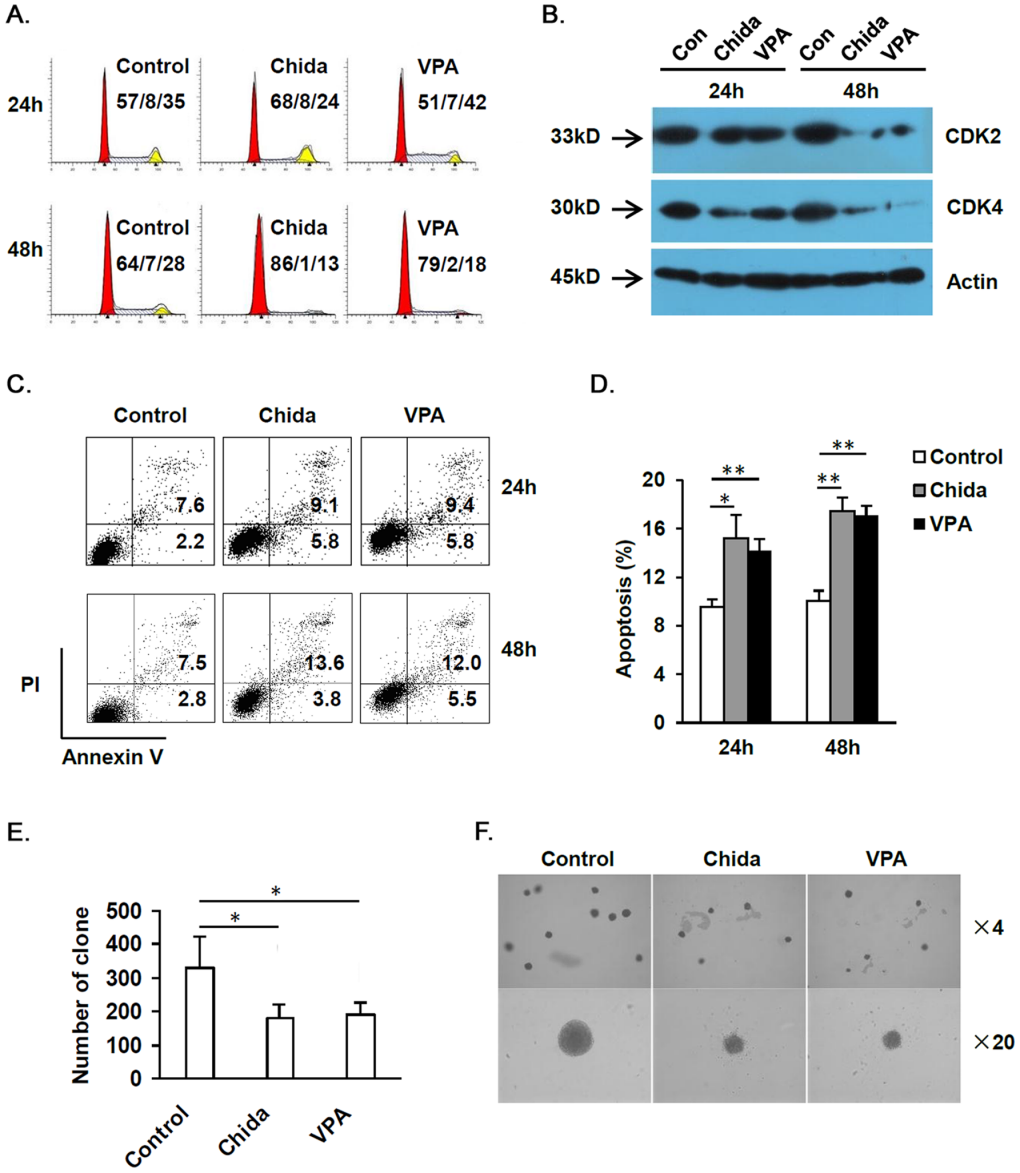
HDAC inhibition induces cell cycle arrest and reduces colony forming ability in AML cells. THP-1 cells were treated with chidamide at 1 µM or VPA at 1 mM for 24 h or 48 h. A. Cell cycle of THP-1 cells was determined by FACS analysis based on DNA content. Cell cycle was presented as percentage of cells in G1/S/G2&M phase. B. Western blot analysis on CDK2 and CDK4. C, D. Apoptosis of THP-1 cells after chidamide or VPA treatment. E, F. Colony forming analysis of THP-1 cells. Number of clone (≥50 cells) per well (E) as well as representative photographs in each group (F) were shown. *P<0.05, **P<0.01.

As PRAME promotes tumor growth via inhibition of retinoic acid induced differentiation and apoptosis, increased PRAME expression might cause accelerated leukemia cell growth and apoptosis resistance. To exclude the possibility that HDAC inhibition may stimulate AML cell growth via increasing PRAME expression, we compared cell cycle, apoptosis and colony forming in THP-1 cells before and after treatment with chidamide (1 µM) or VPA (1 mM). After either chidamide or VPA treatment for 24 h, percentage of THP-1 cells in S phase was not altered, whereas prolonged treatment (48 h) with either drug caused significantly reduced percentage of THP-1 cells in S phase. Percentage of THP-1 cells in G2/M phase was also reduced after treatment with chidamide or VPA for 48 h, though the differences were not statistically significant (Figure 2A and Figure S2A, B). Moreover, both CDK2 and CDK4 were reduced after either chidamide or VPA treatment for 48 h, further supporting cell cycle arrest after prolonged HDAC inhibition (Figure 2B, Figure S2C, D, and see Figure S6C, D and E for representative raw data). Chidamide or VPA treatment for 24 h or 48 h caused significantly increased apoptosis in THP-1 cells (Figure 2C, D). Both chidamide and VPA caused significantly decreased colony forming by THP-1 cells (Figure 2E, F). Thus, AML cell growth was inhibited rather than stimulated after HDAC inhibition, though PRAME expression was increased.

**Figure 3:**
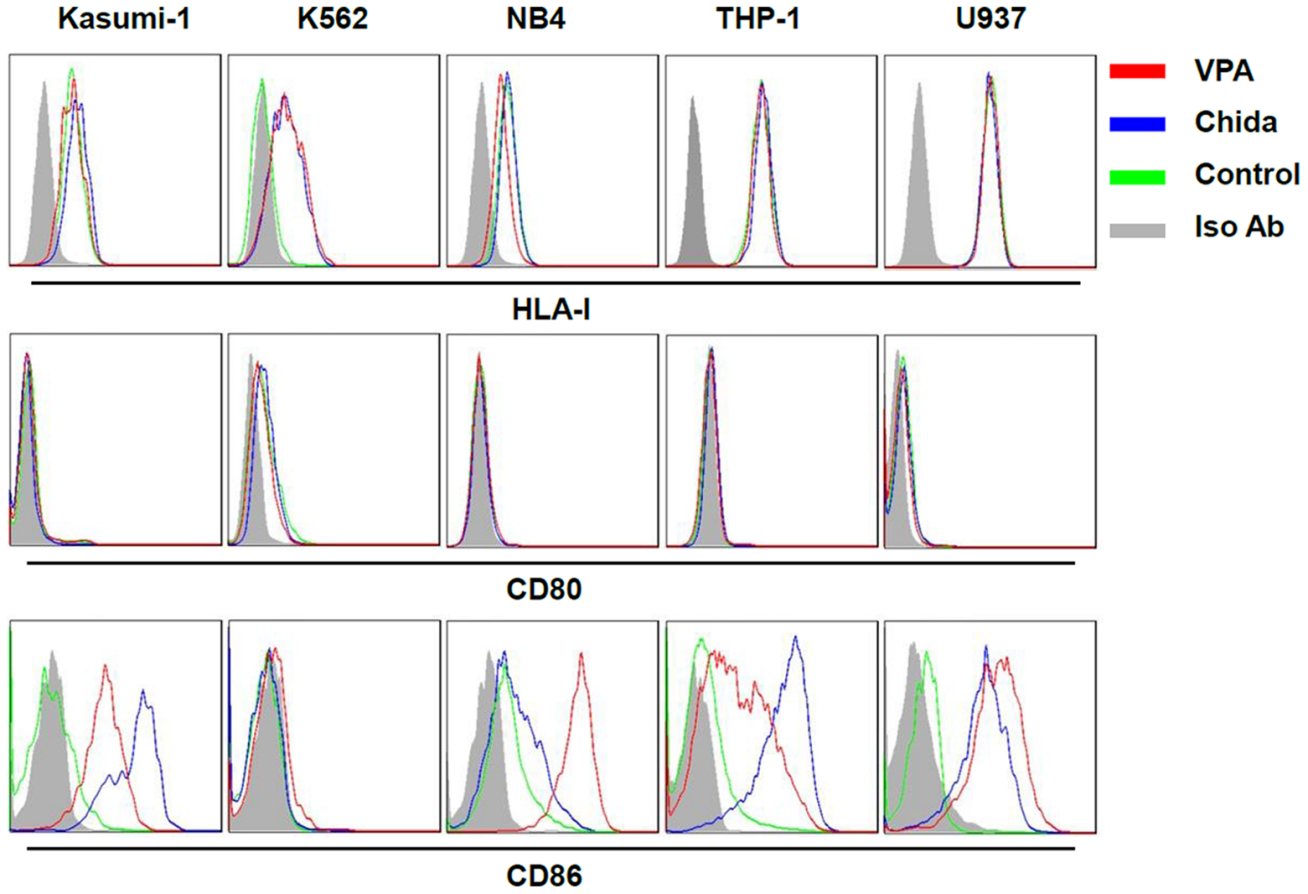
HDAC inhibition upregulates CD86 expression in AML cell lines. AML cells were treated with chidamide (1 µM) or VPA (1 mM) for 24 h. Cell surface expression of HLA-I, CD80, and CD86 were analyzed by FACS analysis.

We further analyzed HLA-I, CD80 and CD86 expression that are directly associated with antigen presentation by AML cells after treatment with chidamide or VPA by flow cytometry analysis. HDAC inhibition drastically upregulated CD86 but not HLA-I or CD80 expression on AML cells except for K562 cells (Figure 3).

### Chidamide Increases PRAME-specific CTL Killing of HLA-A0201^+^ AML Cell Line THP-1

**Figure 4:**
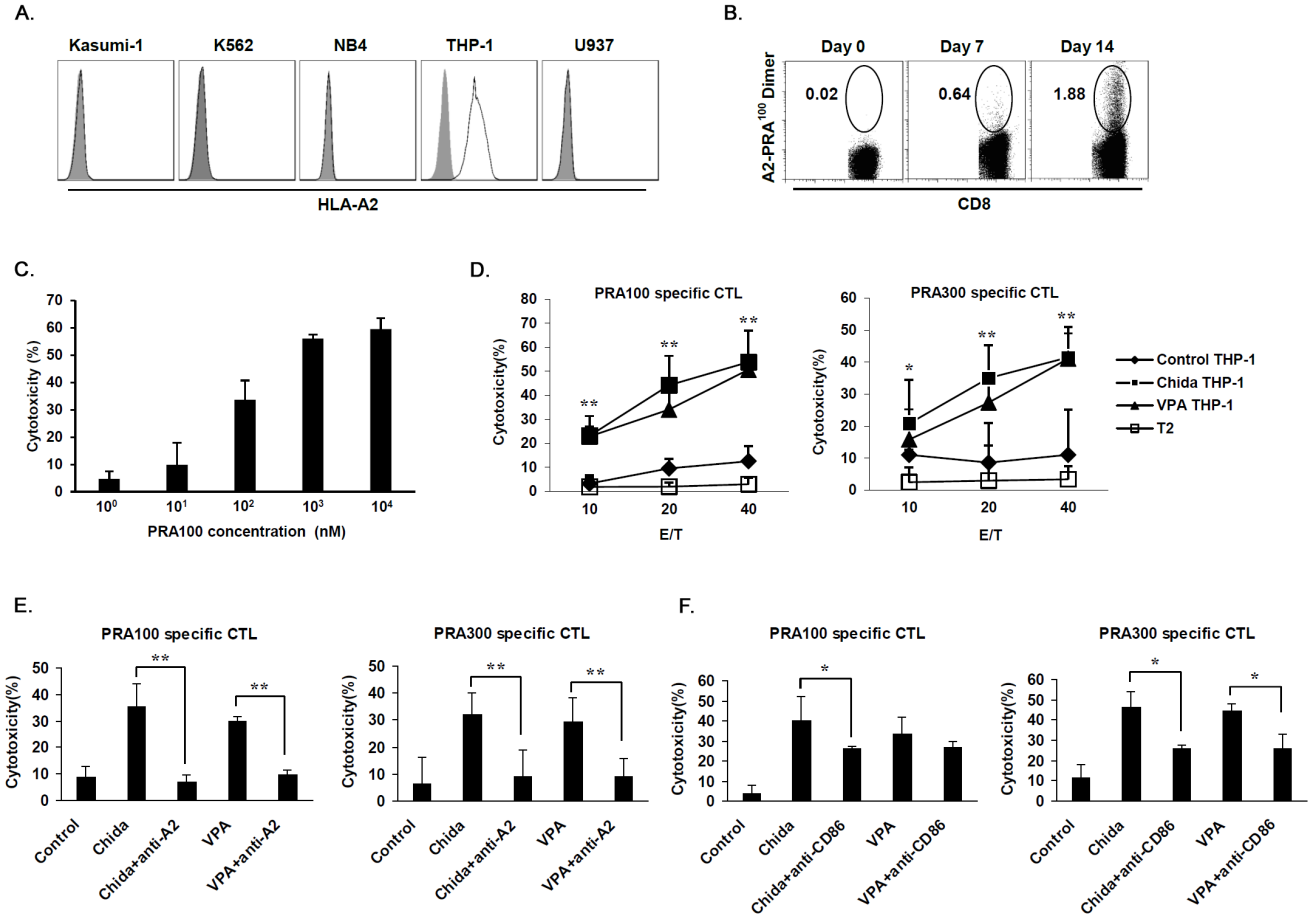
Increased PRAME antigen-specific CTL killing of AML cells after treatment with chidamide. A. AML cell lines were analyzed for HLA-A2 expression by FACS. In the tested AML cell lines, only THP-1 cells were HLA-A2^+^. We confirmed that THP-1 cells were positive for HLA-A0201 allele by DNA sequencing (data not shown). B. Expansion of HLA-A0201-PRA^100–108^ specific CTLs. Number shows the percentage of CD8^+^HLA-A0201-PRA^100–108^ positive cells in CD8^+^ T cells. C. Killing of T2 cells pulsed with PRA^100–108^ at titration concentrations by PRA^100–108^ specific CTLs. D. THP-1 cells treated with chidamide or VPA were analyzed for their sensitivity to CTLs specific for PRA^100–108^ or PRA^300–309^ at various E/T ratios. T2 cells that were not pulsed with peptide were used as negative control targets. E. Blockade of HLA-A2 abrogated cytotoxicity of THP-1 cells by PRAME-specific CTLs. F. Blockade of CD86 significantly reduced cytotoxicity of THP-1 cells by PRAME-specific CTLs. *P<0.05, **P<0.01.

We next asked whether upregulation in PRAME expression could facilitate CTL killing of AML cells. We induced HLA-A0201-restricted PRAME-specific CTLs *in vitro* from PBMCs of 2 HLA-A0201^+^ healthy donors by using an established method [33]. Based on published data and our analysis, only THP-1 was HLA-A0201^+^ in all 5 AML cell lines we used (Figure 4A). DNA sequence analysis confirmed that THP-1 was HLA-A0201^+^ (data not shown). We used THP-1 cells as target cells for specific CTL activity test.

After 2 cycles of stimulation at weekly interval, the number and percentage of PRAME specific CTLs in PBMC CD8^+^ T cells were increased as was shown by staining with PRAME peptide-loaded HLA-A0201 dimer (Figure 4B). CTLs specific for HLA-A0201-restricted PRA^100–108^ or PRA^300–309^ epitope were FACS sorted, followed by a quick expansion and activation procedure. We used T2 cells pulsed with PRA^100–108^ at concentrations ranged from 1 nM to 10,000 nM as target cells to testify the antigen specificity of PRA^100–108^ specific CTLs. As shown in Figure 4C, cytotoxicity of PRA^100–108^ loading T2 cells was increased with the elevation of peptide loading concentration (See Figure S7 for representative raw data). Moreover, cold target inhibition experiments showed significant inhibition of cytotoxicity against chidamide treated THP-1 cells by PRA^100–108^ pulsed T2 cells at 30∶1 and 10∶1 cold to hot target ratios (Figure S3A and see Figure S8 for representative raw data). In order to prove recognition of endogenously processed and presented PRAME by PRAME-specific CTLs, we transfected full length PRAME coding sequence into a HLA-A0201^+^PRAME^−^ cell line SW480 [10]. Cytotoxicity against PRAME transfected SW480 cells was significantly increased as compared with empty vector transfected cells (Figure S3B and see Figure S9 for representative raw data).

We used these activated CTLs to kill chidamide treated or untreated THP-1 cells. Significant increase in cytotoxicity of chidamide treated THP-1 cells by either PRA^100–108^ or PRA^300–309^ specific CTLs was observed when compared with that of non-treated THP-1 cells (Figure 4D). Killing of unpulsed T2 cells was minimal at all E/T ratios (Figure 4D). In parallel with increased cytotoxicity, intracellular staining showed higher percentage of CTLs expressing IFN-γ and TNF-α following coculture with chidamide treated versus non-treated THP-1 cells (Figure S4). Cytotoxicity was reduced to the baseline level when anti-HLA-A2 blocking antibody was supplemented into the coculture system, further supporting an antigen-specific killing by PRAME specific CTLs through a TCR-dependent signaling (Figure 4E). We also used anti-CD86 blocking antibody to determine the possible contribution from CD86 upregulation in CTL cytotoxicity in our experiments. Blockade of CD86 signaling partially impaired CTL killing of THP-1 cells, indicating that increase in CD86 expression induced by HDAC inhibition participated in observed anti-leukemia immune response (Figure 4F).

### Combined Treatment with Chidamide and Decitabine Further Augments PRAME Expression and PRAME-specific CTL Killing of THP-1 Cells

**Figure 5:**
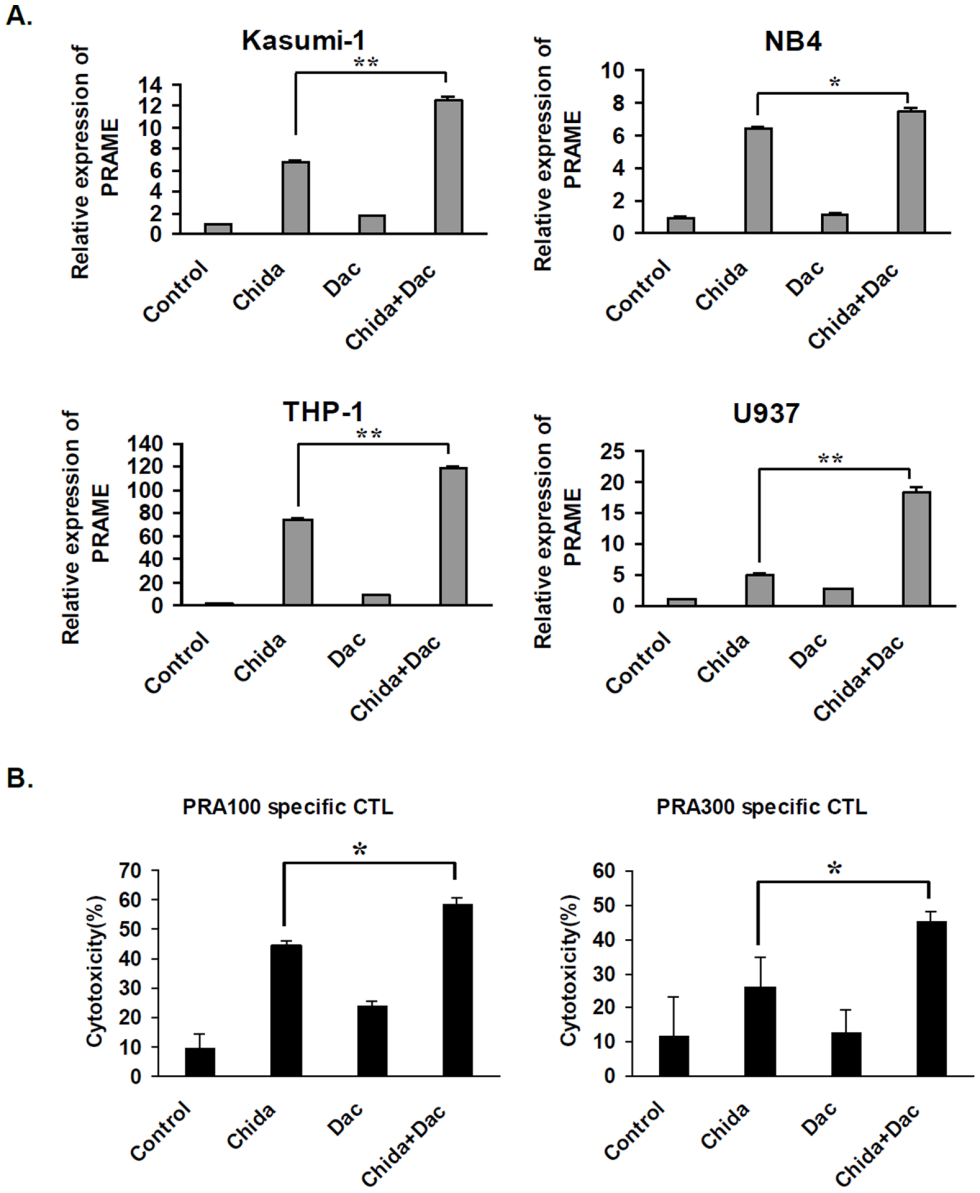
Decitabine further enhances chidamide-induced PRAME upregulation and PRAME antigen-specific CTL cytotoxicity of THP-1 cells. A. AML cell lines were treated with chidamde (1 µM), decitabine (Dac, 0.25 mM), or chidamide/decitabine. PRAME mRNA expression was analyzed by RT-qPCR. Y axis shows folds of PRAME mRNA expression relative to that of non-treated cells. B. THP-1 cells were treated with chidamde, decitabine, or chidamide/decitabine, followed by cytotoxicity analysis with PRAME specific CTLs. *P <0.05, **P<0.01.

PRAME has been reported to be epigenetically regulated by the DNA methylation mechanism, and hypomethylating agents including decitabine and azacitidine upregulate PRAME expression in a variety of tumor cells. We speculated that combined treatment with chidamide and decitabine will jointly upregulate PRAME expression in AML cells. To avoid excessive toxicity of combined treatment with chidamide and decitabine, we used a relatively low concentration of decitabine (0.25 µM) in our study. At such a concentration, decitabine treatment alone caused minimal increase in PRAME mRNA in 4 AML cell lines tested. When decitabine was used in combination with chidamide, significant increase in PRAME expression was observed in all 4 AML cell lines as compared with chidamide treatment alone (Figure 5A), though neither chidamide alone nor combined treatment with decitabine significantly increase PRAME expression in K562 cells or in bone marrow cells from patient #5 in Table 1 (Figure S5). Combined treatment of THP-1 cells with chidamide and decitabine significantly increased cytotoxicity by either PRA^100–108^ or PRA^300–309^ specific CTLs, as compared to that of chidamide treatment alone (Figure 5B). These data demonstrated that combined decitabine treatment with chidamide further significantly enhanced the specific CTL killing of AML cells.

**Table 1:** Information of AML patients.

Patient No.	Gender	Age (year)	Diagnosis	Disease Status	BM Blast
1#	F	63	AML-M2	Chemotherapy-refractory	15.0%
2#	M	50	AML-M4	Not treated	25.7%
3#	M	40	AML-M4	Not treated	75.0%
4#	F	20	AML-M5	NR after chemotherapy	58.8%
5#	F	60	AML-M4	Not treated	50.8%
6#	F	61	AML-M5	Not treated	68.4%

### Chidamide and/or Decitabine Treatment does not Impair CTL Cytotoxic Functions and Chidamide Inhibits Proliferation of Activated T cells

**Figure 6:**
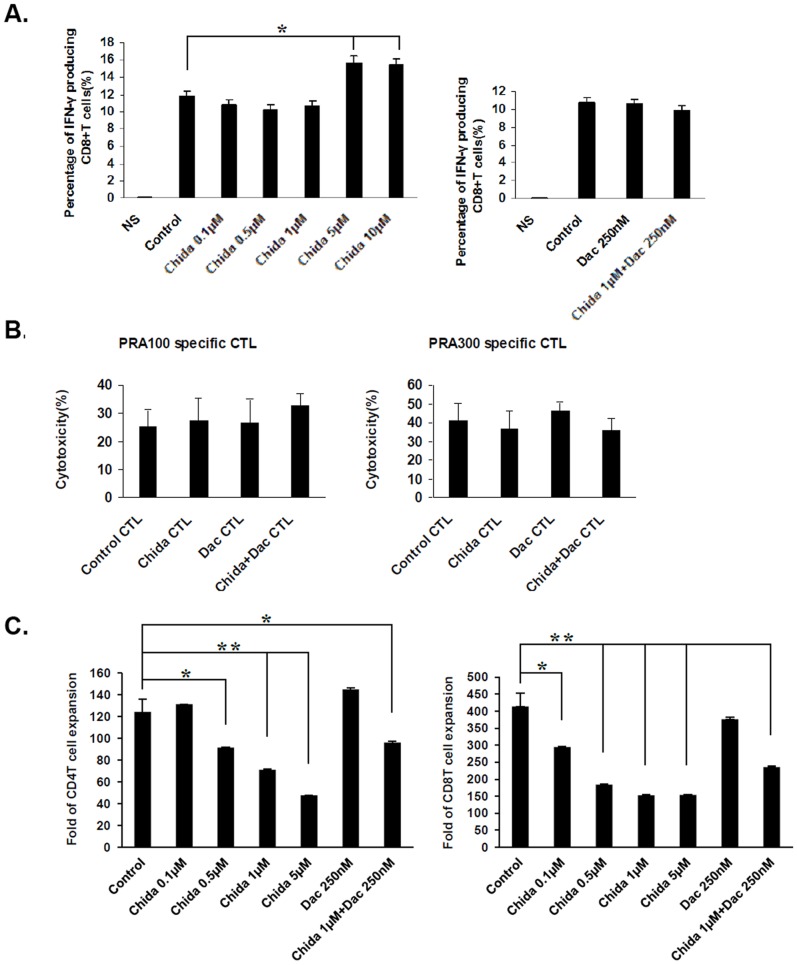
Chidamide and/or decitabine treatment does not impair CTL cytotoxic functions and chidamide inhibits proliferation of activated T cells. A. Percentage of IFN-γ producing CD8^+^ T cells activated by PMA/Ionomycin following treatment with chidamide at various concentrations for 24 h (left), decitabine (Dac), or chidamide/decitabine (right). B. PRAME antigen-specific killing of chidamide treated THP-1 cells by chidamide, decitabine or chidamide/decitabine treated CTLs. C. PBMCs from healthy donors were treated with chidamide, decitabine or chidamide/decitabine at the indicated concentrations. Cells were washed in PBS to remove any residual drugs and suspended in fresh medium, followed by activation with anti-CD3 and anti-CD28 mAbs (1 µg/ml each) at the presence of recombinant human IL-2 (50 IU/ml). PBMCs cultured with only IL-2 were used as control. On day7 of culture, cells in each well were stained with anti-CD8 FITC and anti-CD3 PE, suspended in 500 µl PBS and acquired in flow cytometer for 30 seconds. Dead cells were excluded by gating out high side scatter cells, and number of CD4^+^ T cells (CD3^+^CD8^−^) and CD8^+^ T cells (CD3^+^CD8^+^) were divided by that of control CD4^+^ or CD8^+^ T cells to obtain expansion folds. For combined treatment with chidamide and decitabine in A and B, CTLs were treated with chidamide at 1 µM for 24 h, in combination to decitabine (250 nM) supplemented in culture media twice for 48 h at 24 h interval. In B, chidamide treated THP-1 cells (1 µM for 24 h) were used as target cells, and an E/T ratio of 20/1 was used. *P<0.05, **P<0.01.

When administered *in vivo*, chidamide and decitabine may not only exert their epigenetic regulatory roles on leukemia cells, but also have impact directly on CTL functions. We investigated the possible impact of chidamide and decitabine on CTL cytotoxic functions and proliferation *in vitro*. PBMCs from healthy donors were prepared and treated with chidamide at various concentrations, decitabine (0.25 µM), or chidamide (1 µM) in combination with decitabine (0.25 µM), followed by non-specific activation of T cells by phorbol myristate acetate (PMA) and Ionomycin for IFN-γ intracellular staining analysis or by anti-CD3 and anti-CD28 mAbs for proliferation analysis. We used intracellular FACS staining to analyze IFN-γ expression in CD8^+^ T cells. Our data showed that chidamide had no notable impact on IFN-γ expression in CD8^+^ T cells at concentrations ranged from 0.01 to 1 µM. Slightly increased IFN-γ expression was observed after treatment with chidamide at high concentrations of 5 or 10 µM. Decitabine (0.25 µM) treatment alone or in combination with chidamide (1 µM) had no impact on IFN-γ expression in CD8^+^ T cells (Figure 6A). We also treated PRAME specific CTLs with chidamide and/or decitabine, followed by cytotoxicity analysis against chidamide treated THP-1 cells. CTL cytotoxicity functions remained untouched after treatment with chidamide, decitabine or combination of both (Figure 6B). However, pretreatment with chidamide significantly inhibited the proliferation of CD4^+^ T cells at concentrations ranged from 0.5 to 5 µM and that of CD8^+^ T cells at concentrations ranged from 0.1 to 5 µM (Figure 6C). Pretreatment with decitabine alone at 0.25 µM for 48 h did not significantly affect the proliferation of CD4^+^ T cells or CD8^+^ T cells (Figure 6C).

## Discussion

PRAME is over expressed in a notable proportion of hematological and solid tumor cells but not or minimally expressed in normal tissues except for germ line tissues such as the testis [1], [10]. Accumulating data have demonstrated that PRAME-expressing tumor cells are targets for T cell clones that express PRAME epitope-specific TCRs [7], [8], [9], [11], [12]. We confirmed the tumor-exclusive expression pattern of PRAME by showing that PRAME mRNA was detected in AML and ALL cell lines as well as in bone marrow from AML patients but not in bone marrow or peripheral blood from healthy donors. We also reproduced an antigen-specific killing of AML cells by purified CTLs that recognize HLA-A0201-restricted PRAME epitopes. Thus, PRAME remains a candidate target for both active and adoptive immunotherapy whilst much effort shall be taken to justify its therapeutic potential.

PRAME inhibits retinoic acid induced differentiation and apoptosis in leukemia cells [4], [35]. Therefore, PRAME expressing leukemia cells may have growth advantage over PRAME negative leukemia cells. In our study, HDAC inhibition induced upregulation of PRAME did not result in accelerated cell growth in AML cells. This could be explained by the well-recognized anti-tumor role of HDAC inhibitors via inducing cell cycle arrest and apoptosis [14], [18]. In a clinical setting, the relationship between PRAME expression level and prognosis in patients with hematological or solid malignancies has been controversial [36], [37], [38], [39], [40]. This is not surprising, as PRAME expression level is only one factor among enormous other factors that may influence the clinical outcomes in these patients. A possible explanation to this discrepancy comes from the perspective of adaptive anti-tumor immunity, where PRAME expressing leukemia cells are prone to be attacked by T cells that specifically recognize PRAME epitopes presented by autologous HLA-I molecules on leukemia cell surface [11], [13].

Though PRAME expression is detected in a notable fraction of leukemia samples, PRAME specific CTL response is not detectable or at a very low level in most leukemia patients. These data suggested a ‘threshold’ of PRAME expression that is required (but may not be adequate) for the activation of CTL immune response, which was supported by that the T cell response to multiple PRAME epitopes was more likely to be detected in patients with PRAME expression level of over 0.001 than those less than 0.001 [8], [13]. This hypothesis pointed to a possible future therapeutic paradigm that epigenetic modulator such as HDAC inhibitor may activate autologous and adoptive anti-leukemia CTL responses through increasing CTA expression [26]. Such an idea is supported by our study results showing a correlation between PRAME expression level and PRAME antigen-specific killing of AML cells by CTLs *in vitro*, as well as by a few studies reporting increased PRAME specific CTL killing of malignant cells after DNA demethylation induced PRAME expression *in vitro*, and more importantly, increased MAGE-A1 specific CTL response in AML and MDS patients following combined treatment with VPA and AZA [8], [10], [12], [25].

CTLs generated in our study were specific for PRAME peptide presented by HLA-A0201, as demonstrated by increasing cytotoxicity against T2 cells pulsed with PRAME peptide at increasing titration concentrations. This is also supported by cold target inhibition experiment results showing inhibition of cytotoxicity against chidamide treated THP-1 (hot targets) by PRAME peptide pulsed T2 cells (cold targets), as well as that blockade of HLA-A2 reduced CTL killing to a background level. Moreover, cytotoxicity against PRAME vector transfected SW480 cells that were HLA-A0201^+^PRAME^−^ before transfection was significantly higher that of empty vector transfected cells, indicating specific recognition of endogenously processed and presented PRAME by CTLs [10]. CD86 provides costimulatory signals in the immune synapse between CTLs and their target cells [41], [42]. In our study, antibody blockade of CD86 significantly reduced CTL killing of THP-1 cells, suggesting a contributing role of CD86 costimulation. However, PRAME antigen-specific CTL killing of chidamide treated THP-1 cells at the presence of anti-CD86 blocking antibody remained significantly higher than that of untreated THP-1 cells. This could be explained by that the effector CD8^+^ T cells form immune synapse with target cells presenting cognate MHC-peptide complex in the absence of costimulation signals [43].

We observed upregulation of PRAME in the mRNA level after treatment with chidamide or VPA, two HDAC inhibitors. Our data was in accordance with previous study results showing epigenetic upregulation of CTA expression following HDAC inhibition in human and mouse tumor cells [6], [22], [25]. The generally accepted histone remodeling mechanism may at least partially explain these data, whereas the detailed mechanisms underlying the CTA stimulating role by HDAC inhibition is not fully understood [14]. In our study, increased PRAME mRNA expression after chidamide treatment was observed in AML and ALL cell lines but not in normal bone marrow or peripheral blood cells. Accordingly, we observed increased aceH3 in AML cells after HDAC inhibition, indicating that HDAC inhibition may upregulate PRAME expression via a chromatin remodeling mechanism. In parallel with increased PRAME mRNA, we observed increased PRAME protein expression in THP-1 cells after chidamide or VPA treatment *in vitro*. Our study results were supported by a previous study on another cancer testis antigen (melanoma associated antigens, MAGE), in which upregulation of MAGE-A1 mRNA expression was associated with increase in MAGE-A1 protein [25]. After treatment with chidamide, PRAME mRNA expression in THP-1 cells was increased for at least 1 week. Given that chidamide is continuously administered orally in a phase II clinical trial in patients with peripheral T-cell lymphoma (PTCL) or cutaneous T-cell lymphoma (CTCL) (not published data), we speculate that continuous chidamide administration may keep a prolonged upregulation of PRAME, facilitating the activation, proliferation, differentiation, recognition, and killing of AML cells by PRAME-specific T cells. In our experiment, PRAME mRNA was not detected in bone marrow or peripheral blood cells from healthy donors before or after chidamide treatment *in vitro*. However, it remains possible that chidamide treatment *in vivo* may stimulate PRAME expression in other normal cell types, resulting in cytotoxicity of normal tissues by PRAME antigen specific CTLs. Further study is required to evaluate possible impact of HDAC inhibitors on PRAME expression in normal tissues.

PRAME expression is regulated by DNA methylation mechanisms [3], [6], [10], [12]. In previous studies, relative high concentrations of decitabine or AZA were used (≥1 µM) [10], [12]. Although there were dose-dependent effects, the concentration of decitabine used in these experiments was higher than the optimal demethylation concentration of decitabine *in vitro* (0.1–1 µM) [44]. In our study, we used 0.25 µM of decitabine treatment in combination with chidamide, in order to minimize direct cytotoxicity of AML cells. Though decitabine at 0.25 µM had minimal boost effect on PRAME mRNA expression in AML cells, it significantly increased PRAME expression when used in combination with chidamide. Decitabine at 0.25 µM also significantly increased VPA induced upregulation of PRAME in AML cell lines (data not shown). Our data suggested a synergistic effect between HDAC inhibition and DNA demethylation in the upregulation of PRAME in AML cell lines.

Epigenetic modulators including HDAC inhibitors and hypomethylating agents also exert direct influence on immune cell functions both *in vitro* and *in vivo* [45], [46], [47], [48]. For instance, HDAC inhibitor Trichostatin A (TsA) has been reported to promote both primary and recall CD8^+^ T cell responses in mouse models of infectious diseases [45], [47], [49]. In contrast to this immune-stimulating role, other HDAC inhibitors such as SAHA and ITF2357 reduce mouse graft-versus-host disease (GVHD) via suppressing proinflammatory functions of dendritic cells [48]. Hypomethylating agents decitabine and azacitidine have been reported to induce generation of Foxp3^+^ regulatory T cells *in vivo* in a mouse GVHD model [46]. In our study, chidamide did not influence IFN-γ expression by non-specifically activated CD8^+^ T cells at concentrations of ≤1 µM. At higher concentrations (5 or 10 µM), chidamide slightly increased IFN-γ expression by CD8^+^ T cells. Treatment of CTLs with chidamide alone or in combination with decitabine did not alter PRAME specific CTL killing of chidamide treated THP-1 cells. Our study results are supported by data from another group in which neither SAHA nor TsA had direct influence on human T cell cytotoxicity *in vitro* [50]. However, pretreatment with chidamide but not decitabine significantly inhibited the proliferation of both CD4^+^ and CD8^+^ T cells activated by anti-CD3 and anti-CD28 mAbs, suggesting a possible inhibition of anti-leukemia T cell immunity by chidamide *in vivo*. Therefore, further studies are required to explore the possible impact of chidamide on anti-tumor T cell immune response in mouse models and patients with malignancies in whom chidamide treatment is indicated.

In conclusion, we demonstrated that *in vitro* treatment with subtype-selective HDAC inhibitor chidamide alone or in combination with decitabine increased PRAME antigen-specific cytotoxicity of CTL through upregulation of PRAME and CD86 expression in AML cell lines. Our study results added a new set of data to the hypothesis that epigenetic modulators may improve the effects of anti-leukemia immunotherapy via increasing the immunogenicity of leukemia cells.

## Supporting Information

Figure S1 **Chidamide does not induce PRAME mRNA expression in normal blood and bone marrow cells.** Chidamide treated (1 µM) or non-treated mononuclear cells from peripheral blood (PB) and bone marrow (BM) of 2 healthy donors were cultured in RPMI 1640 supplemented with 10% FBS at 37°C in a CO_2_ incubator for 48 h. Cells were washed and harvested, followed by RT-PCR analysis of PRAME mRNA expression (35 PCR cycles). GAPDH was used as internal control (26 PCR cycles). K562 cells were used as a positive control for PRAME. PRAME mRNA was not detected in either non-treated or chidamide treated normal PB or BM mononuclear cells.
(TIF)

Figure S2 **Statistics of cell cycle analysis and image density of CDK2/4 western blot in THP-1 cells following chidamide or VPA treatment** ***in vitro*** **.** A and B. THP-1 cells cultured in 24-well plates in triplicates were non-treated or treated with chidamide (1 µM) or VPA (1 mM) *in vitro* for 24 (A) or 48 h (B). Cells were harvested, followed by FACS analysis of cell cycle based on DNA content. Data are presented as mean±S.D. of percentage of cells in G1, S or G2/M phase. *P<0.05, **P<0.01. C and D. An ImageJ 2.1.4.7 software was used to analyze the image density of western blot staining shown in Figure 2B. Relative density of CDK2 (C) and CDK4 (D) to that of β-actin is shown.
(TIF)

Figure S3 **PRAME antigen-specific cytotoxicity of CTLs.** HLA-A0201-PRA^100–108^ specific CTLs were generated as described in methods. A. Cold target inhibition experiment showed significant inhibition of cytotoxicity against chidamide treated THP-1 cells by PRA^100–108^ pulsed T2 cells as cold targets at 30∶1 and 10∶1 cold to hot target ratios. B. In order to prove recognition of endogenously processed and presented PRAME, we transiently transfected SW480 cells with empty vector or PRAME vector as described in methods, followed by cytotoxicity assay with HLA-A0201-PRA^100–108^ specific CTLs. **P <0.01.
(TIF)

Figure S4 **Increased IFN-γ and TNF-α expression by PRAME specific CTLs induced by chidamide treated THP-1 cells.** HLA-A0201-PRA^100–108^ specific CTLs (responder) were cocultured with X-ray irradiated (16 Gy) non-treated or chidamide treated THP-1 cells for 24 h at a responder/stimulator ratio of 10/1 in triplicates in 24-well plate. Five hours before harvest of cells, Golgistop was added to cell medium. Cells were stained with anti-human CD8, anti-human CD3 and intracellular anti-human IFN-γ or TNF-α, followed by FACS analysis. Representative dot plot (A) and column diagraph with statistical analysis results (B) are shown. *P<0.05.
(TIF)

Figure S5 **Combined treatment with chidamide and decitabine does not increase PRAME mRNA expression in K562 cells or bone marrow cells from patient #5.** THP-1 cells, K562 cells and bone marrow cells from patient #5 in Table 1 were treated with chidamide, decitabine or in combination. RT-PCR was used to analyze PRAME mRNA expression.
(TIF)

Figure S6 **Representative raw data of western blot.** A. The 57 kD bands that represent specific staining of PRAME, as well as non-specific staining (presumably due to the polyclonal antibody) are shown. The specific staining of PRAME is shown as Figure 1D PRAME. B. The 46 kD bands in left panels are shown as Figure 1D actin. C. The bands in right panels (24 h and 48 h) are shown as Figure 2B CDK2. D. The photograph is shown as Figure 2B CDK4. E. The photograph is shown as Figure 2B actin.
(TIF)

Figure S7 **Representative raw data for** **Figure 4C** **.** A. CFSE labeled T2 cells pulsed with PRAME^100–108^ peptide at concentrations ranged 1 to 10,000 nM were cultured alone as spontaneous death control cells. B. Coculture of PRAME^100–108^ specific CD8^+^ T cells with CFSE labeled T2 cells pulsed with PRAME^100–108^ peptide at concentrations ranged 1 to 10,000 nM. Cells were stained with PI. In each group, representative forward scatter/side scatter dot plots, as well as FL-1/FL-3(CFSE/PI) dot plots in triplicate wells are shown. Numbers of viable targets (CFSE^+^PI^−^) and dead targets (CFSE^+^PI^+^) are presented. Results of calculation are shown in the attached table.
(TIF)

Figure S8 **Representative raw data for Figure S3A.** Chidamide treated THP-1 cells (labeled with CFSE) were cultured alone (spontaneous death control) or with PRAME^100–108^ specific CD8^+^ T cells with or without PRAME^100–108^ pulsed T2 cells. Cells were stained with PI. In each group, representative forward scatter/side scatter dot plots, as well as FL-1/FL-3(CFSE/PI) dot plots in triplicate wells are shown. Numbers of viable (CFSE^+^PI^−^) and dead (CFSE^+^PI^+^) THP-1 target cells are presented. Results of calculation and statistical analysis are shown in the attached table.
(TIF)

Figure S9 **Representative raw data for Figure S3B.** Empty vector or PRAME vector transfected SW480 cells were labeled with CFSE, followed by culture alone (spontaneous death control) or with PRAME^100–108^ specific CD8^+^ T cells. Cells were stained with PI. In each group, representative forward scatter/side scatter dot plots, as well as FL-1/FL-3(CFSE/PI) dot plots in triplicate wells are shown. Numbers of viable (CFSE^+^PI^−^) and dead (CFSE^+^PI^+^) SW480 target cells are presented. Results of calculation and statistical analysis are shown in the attached table.
(TIF)
