# Supplementary figures and images for: Increased PRAME-Specific CTL Killing of Acute Myeloid Leukemia Cells by Either a Novel Histone Deacetylase Inhibitor Chidamide Alone or Combined Treatment with Decitabine
Source: PLoS One. 2013 Aug 5;8(8):e70522. doi: 10.1371/journal.pone.0070522 (PMC3734248; doi:10.1371/journal.pone.0070522)

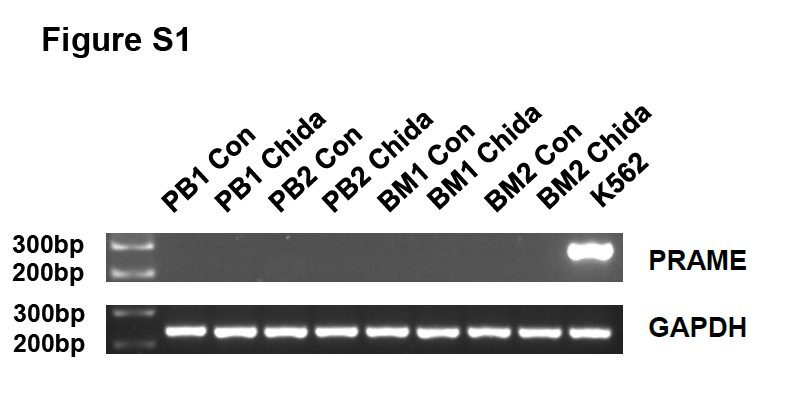

Supplement: Figure S1 — Chidamide does not induce PRAME mRNA expression in normal blood and bone marrow cells. Chidamide treated (1 µM) or non-treated mononuclear cells from peripheral blood (PB) and bone marrow (BM) of 2 healthy donors were cultured in RPMI 1640 supplemented with 10% FBS at 37°C in a CO2 incubator for 48 h. Cells were washed and harvested, followed by RT-PCR analysis of PRAME mRNA expression (35 PCR cycles). GAPDH was used as internal control (26 PCR cycles). K562 cells were used as a positive control for PRAME. PRAME mRNA was not detected in either non-treated or chidamide treated normal PB or BM mononuclear cells. (TIF) [file pone.0070522.s001.tif]

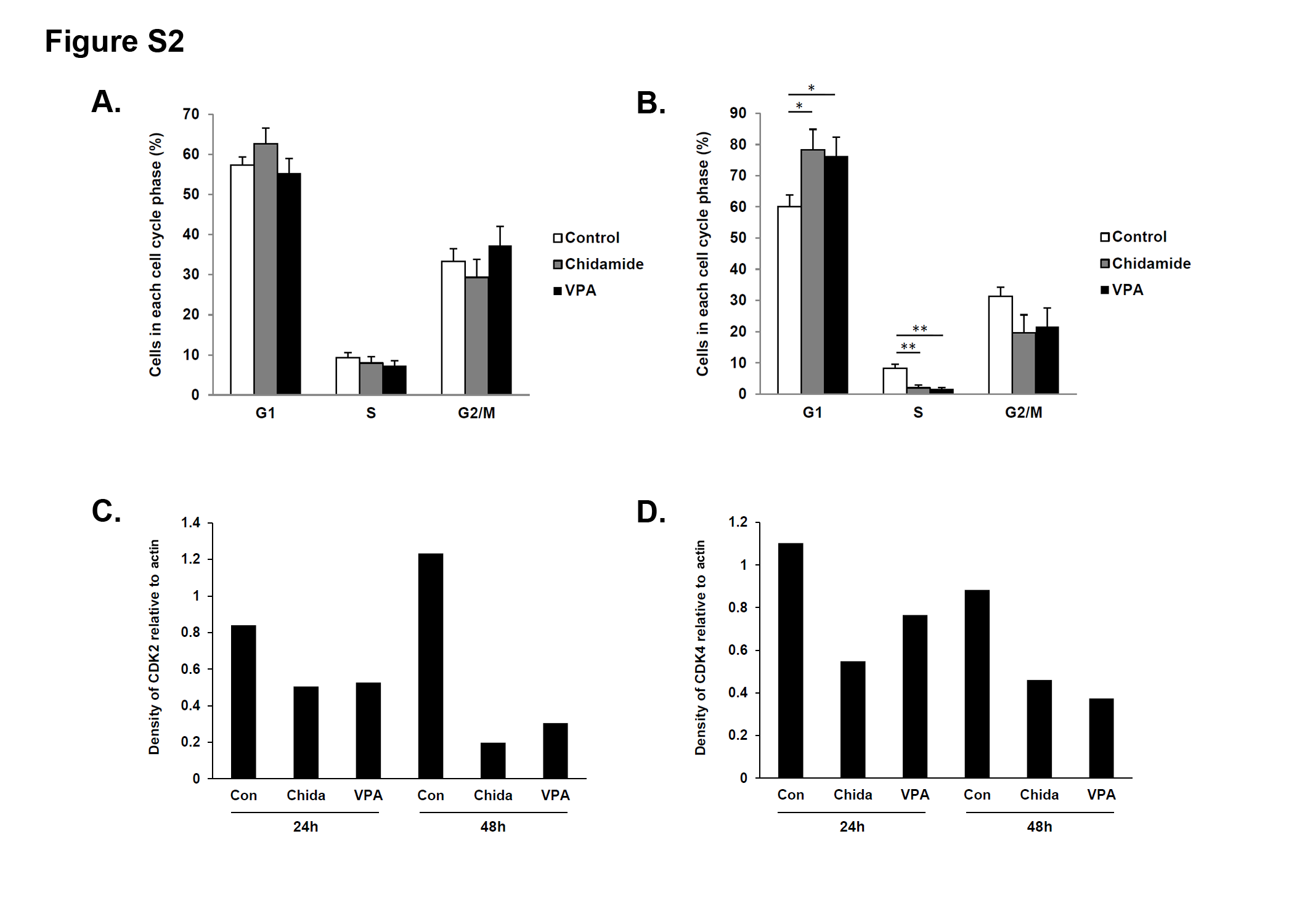

Supplement: Figure S2 — Statistics of cell cycle analysis and image density of CDK2/4 western blot in THP-1 cells following chidamide or VPA treatment in vitro . A and B. THP-1 cells cultured in 24-well plates in triplicates were non-treated or treated with chidamide (1 µM) or VPA (1 mM) in vitro for 24 (A) or 48 h (B). Cells were harvested, followed by FACS analysis of cell cycle based on DNA content. Data are presented as mean±S.D. of percentage of cells in G1, S or G2/M phase. *P<0.05, **P<0.01. C and D. An ImageJ 2.1.4.7 software was used to analyze the image density of western blot staining shown in Figure 2B. Relative density of CDK2 (C) and CDK4 (D) to that of β-actin is shown. (TIF) [file pone.0070522.s002.tif]

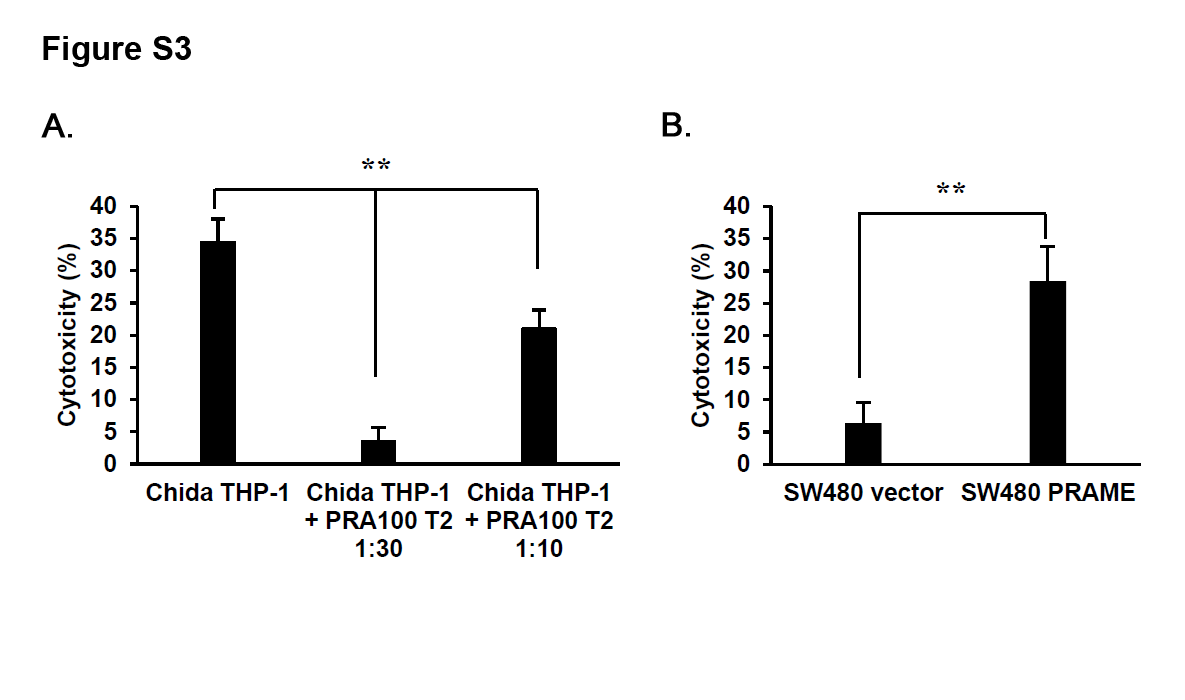

Supplement: Figure S3 — PRAME antigen-specific cytotoxicity of CTLs. HLA-A0201-PRA100–108 specific CTLs were generated as described in methods. A. Cold target inhibition experiment showed significant inhibition of cytotoxicity against chidamide treated THP-1 cells by PRA100–108 pulsed T2 cells as cold targets at 30∶1 and 10∶1 cold to hot target ratios. B. In order to prove recognition of endogenously processed and presented PRAME, we transiently transfected SW480 cells with empty vector or PRAME vector as described in methods, followed by cytotoxicity assay with HLA-A0201-PRA100–108 specific CTLs. **P <0.01. (TIF) [file pone.0070522.s003.tif]

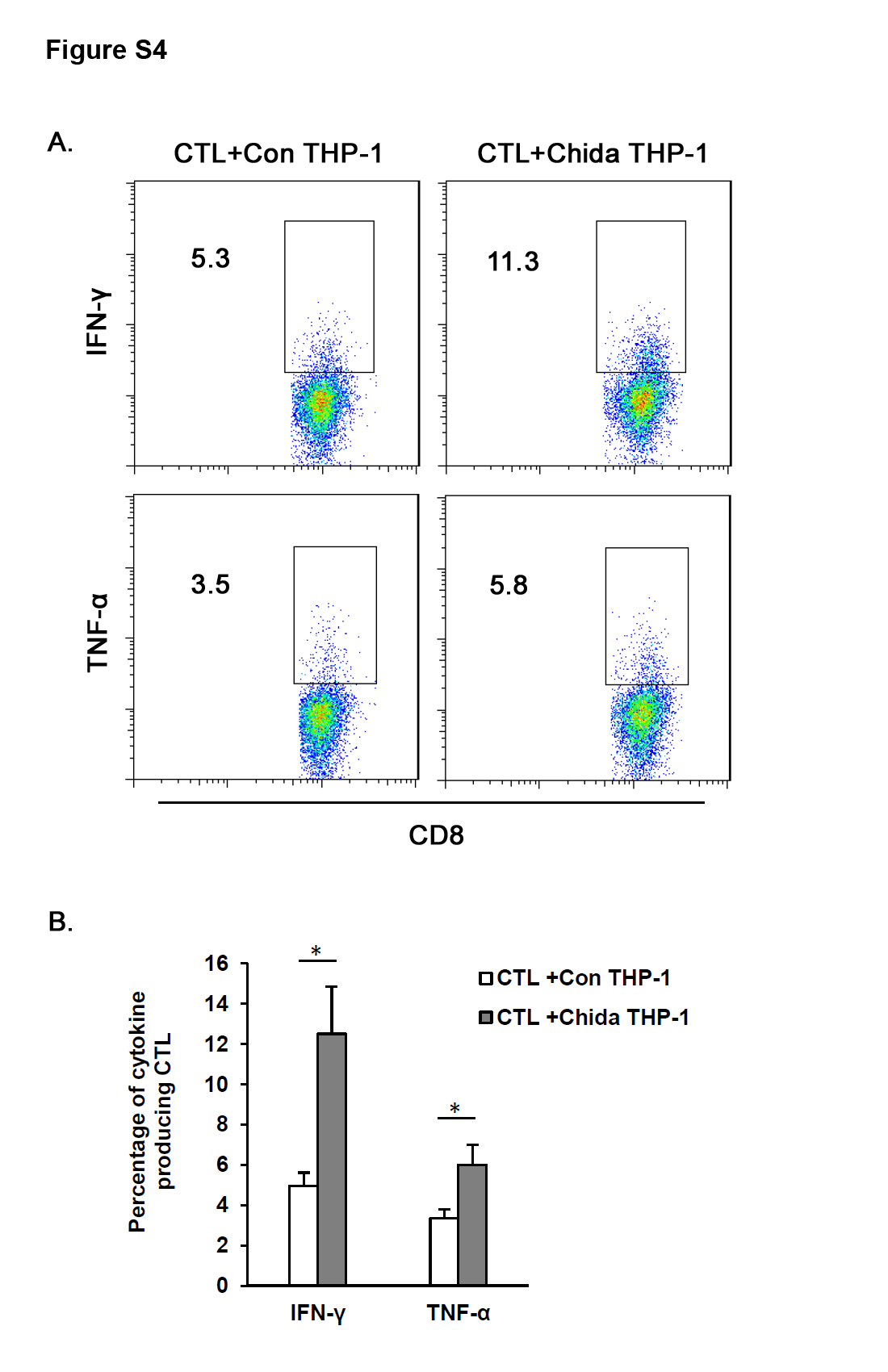

Supplement: Figure S4 — Increased IFN-γ and TNF-α expression by PRAME specific CTLs induced by chidamide treated THP-1 cells. HLA-A0201-PRA100–108 specific CTLs (responder) were cocultured with X-ray irradiated (16 Gy) non-treated or chidamide treated THP-1 cells for 24 h at a responder/stimulator ratio of 10/1 in triplicates in 24-well plate. Five hours before harvest of cells, Golgistop was added to cell medium. Cells were stained with anti-human CD8, anti-human CD3 and intracellular anti-human IFN-γ or TNF-α, followed by FACS analysis. Representative dot plot (A) and column diagraph with statistical analysis results (B) are shown. *P<0.05. (TIF) [file pone.0070522.s004.tif]

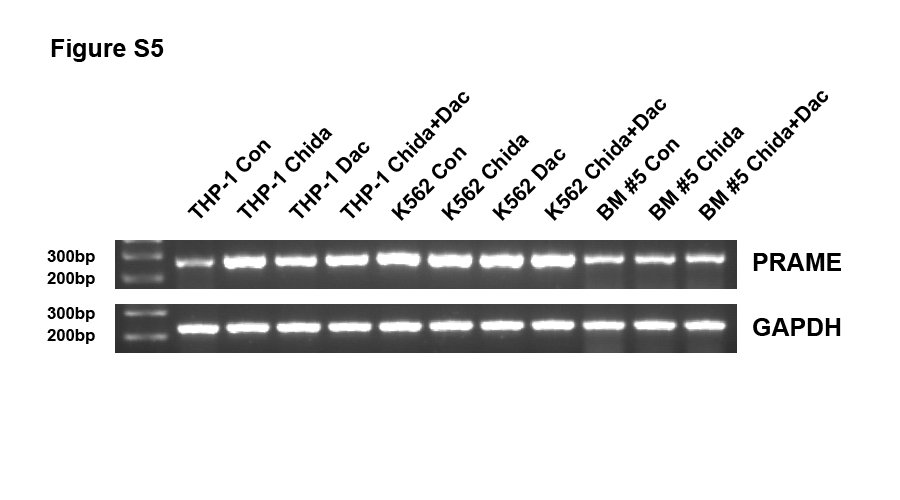

Supplement: Figure S5 — Combined treatment with chidamide and decitabine does not increase PRAME mRNA expression in K562 cells or bone marrow cells from patient #5. THP-1 cells, K562 cells and bone marrow cells from patient #5 in Table 1 were treated with chidamide, decitabine or in combination. RT-PCR was used to analyze PRAME mRNA expression. (TIF) [file pone.0070522.s005.tif]

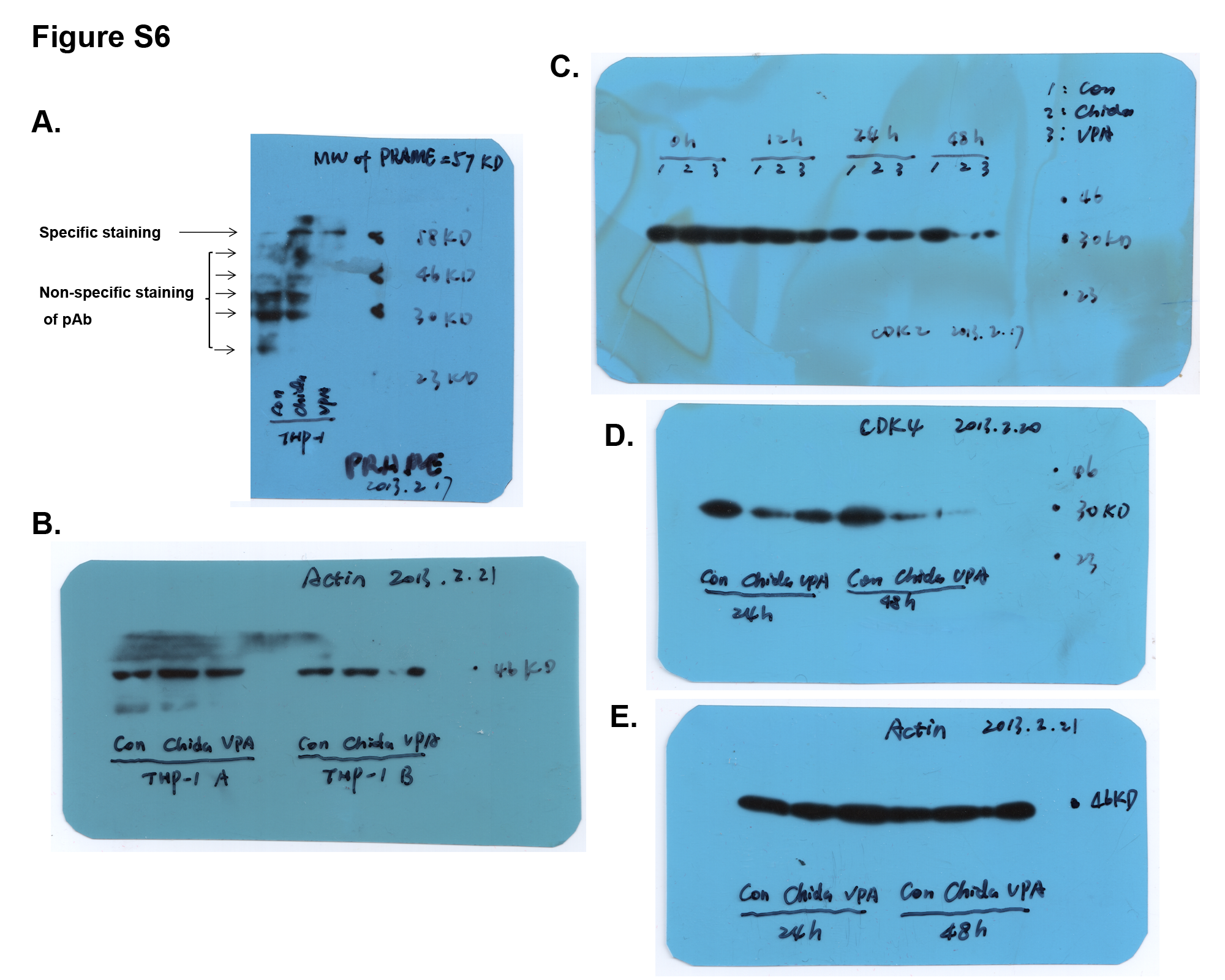

Supplement: Figure S6 — Representative raw data of western blot. A. The 57 kD bands that represent specific staining of PRAME, as well as non-specific staining (presumably due to the polyclonal antibody) are shown. The specific staining of PRAME is shown as Figure 1D PRAME. B. The 46 kD bands in left panels are shown as Figure 1D actin. C. The bands in right panels (24 h and 48 h) are shown as Figure 2B CDK2. D. The photograph is shown as Figure 2B CDK4. E. The photograph is shown as Figure 2B actin. (TIF) [file pone.0070522.s006.tif]

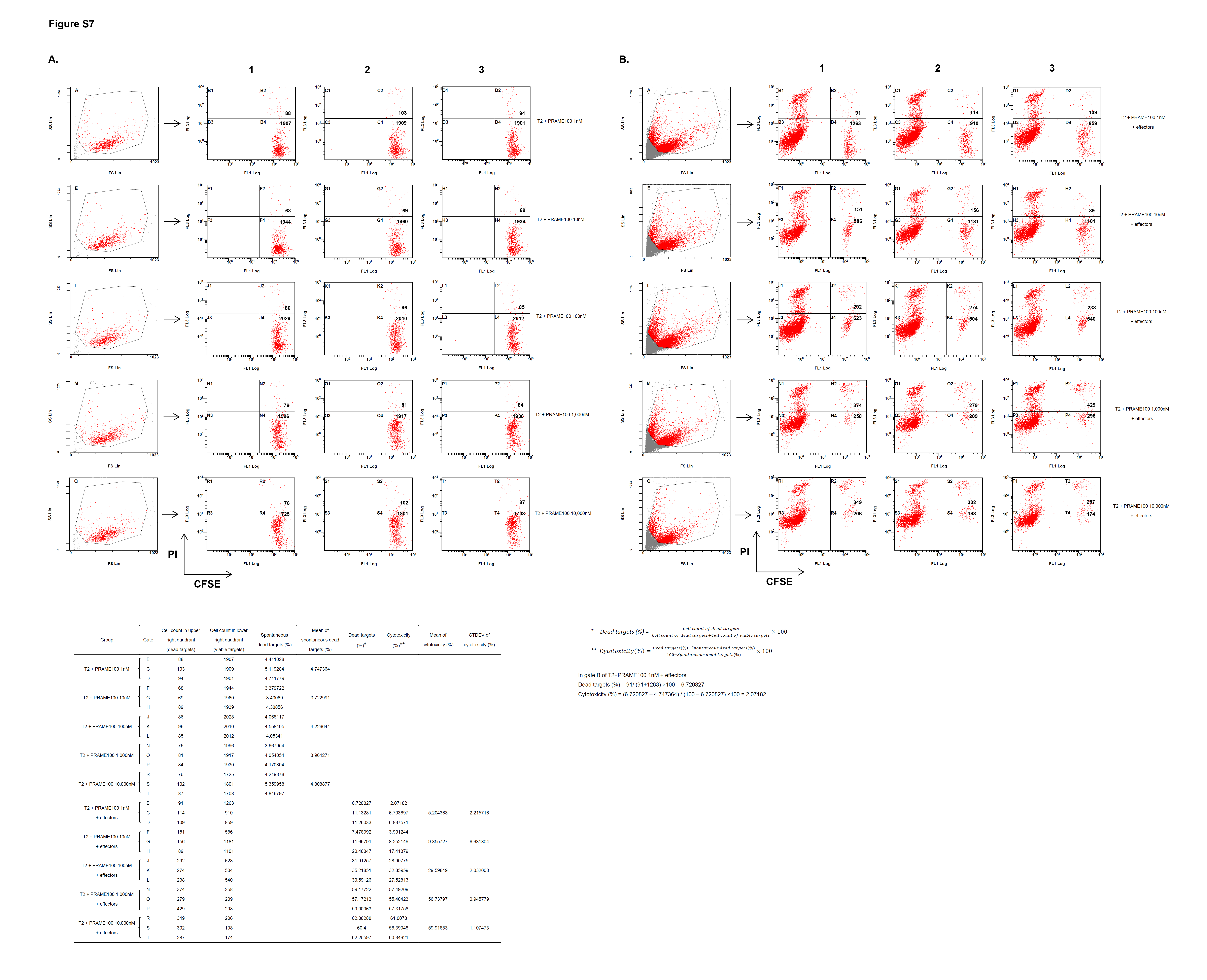

Supplement: Figure S7 — Representative raw data for Figure 4C . A. CFSE labeled T2 cells pulsed with PRAME100–108 peptide at concentrations ranged 1 to 10,000 nM were cultured alone as spontaneous death control cells. B. Coculture of PRAME100–108 specific CD8+ T cells with CFSE labeled T2 cells pulsed with PRAME100–108 peptide at concentrations ranged 1 to 10,000 nM. Cells were stained with PI. In each group, representative forward scatter/side scatter dot plots, as well as FL-1/FL-3(CFSE/PI) dot plots in triplicate wells are shown. Numbers of viable targets (CFSE+PI−) and dead targets (CFSE+PI+) are presented. Results of calculation are shown in the attached table. (TIF) [file pone.0070522.s007.tif]

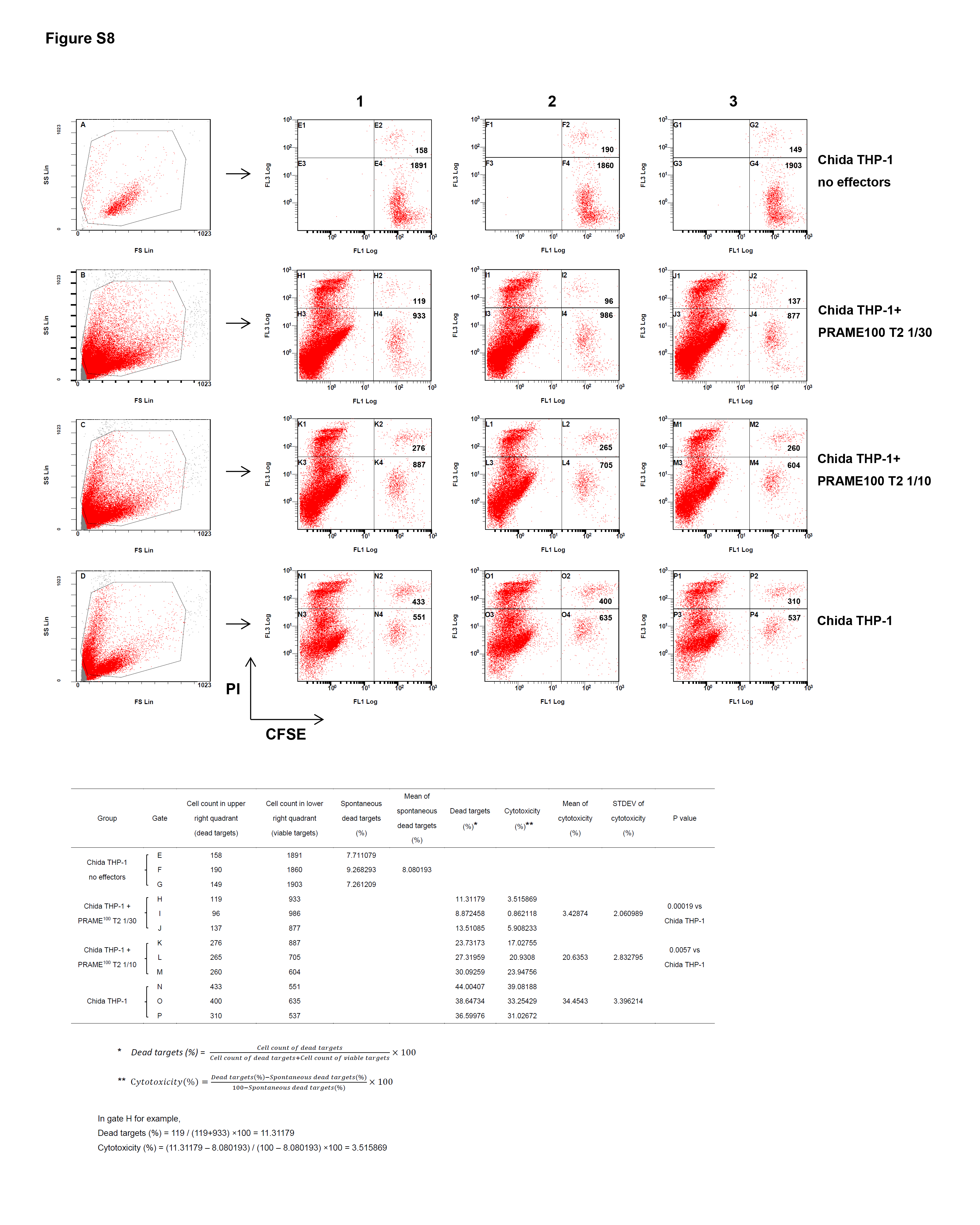

Supplement: Figure S8 — Representative raw data for Figure S3A. Chidamide treated THP-1 cells (labeled with CFSE) were cultured alone (spontaneous death control) or with PRAME100–108 specific CD8+ T cells with or without PRAME100–108 pulsed T2 cells. Cells were stained with PI. In each group, representative forward scatter/side scatter dot plots, as well as FL-1/FL-3(CFSE/PI) dot plots in triplicate wells are shown. Numbers of viable (CFSE+PI−) and dead (CFSE+PI+) THP-1 target cells are presented. Results of calculation and statistical analysis are shown in the attached table. (TIF) [file pone.0070522.s008.tif]

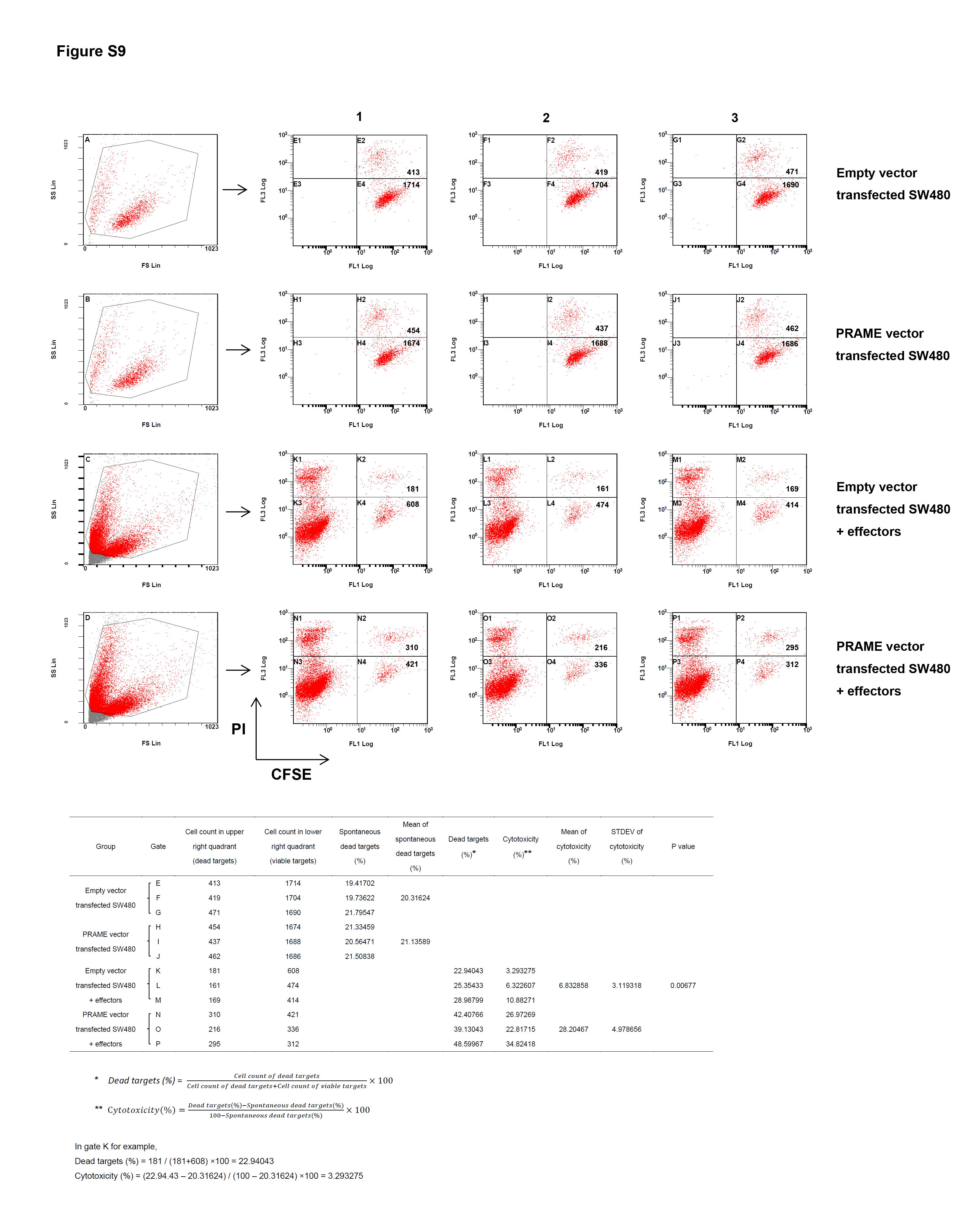

Supplement: Figure S9 — Representative raw data for Figure S3B. Empty vector or PRAME vector transfected SW480 cells were labeled with CFSE, followed by culture alone (spontaneous death control) or with PRAME100–108 specific CD8+ T cells. Cells were stained with PI. In each group, representative forward scatter/side scatter dot plots, as well as FL-1/FL-3(CFSE/PI) dot plots in triplicate wells are shown. Numbers of viable (CFSE+PI−) and dead (CFSE+PI+) SW480 target cells are presented. Results of calculation and statistical analysis are shown in the attached table. (TIF) [file pone.0070522.s009.tif]
